# Supplementary material for: Converging sensory and motor cortical inputs onto the same striatal neurons: An in vivo intracellular investigation
Source: PLoS One. 2020 Feb 5;15(2):e0228260. doi: 10.1371/journal.pone.0228260 (PMC7001913; doi:10.1371/journal.pone.0228260)
Supplement: Attachment — Submitted filename: reviewer comments.pdf [file pone.0228260.s001.pdf]

The paper by Charpier et al describes intracellular recordings from neurons in the dorsolateral striatum of anesthetized rats. The authors show that striatal projection neurons (MSNs) responding to contralateral whisker deflections also respond to electrical stimulation of the ipsilateral motor cortex, thus suggesting that they may be involved in sensorimotor processes. The authors also confirm that these responses to electrical stimulation are monosynaptic, based on the onset latencies in striatal and cortical neurons. The manuscript is interesting, timely, and well-written, and the data presented are convincing. I have just a few comments regarding additional information to be added and the relationship to previously published studies.

Sensorimotor targeting: The targeting of MSNs by sensory and motor cortices, even in overlapping regions, was shown in earlier papers using other methods: (Wall…Kreitzer, Neuron (2013), Reig & Silberberg, Cerebral cortex (2016),). Moreover, it was shown that both types of MSNs (direct and indirect pathway neurons) receive these inputs. The issue of selective targeting of direct and indirect pathways should be addressed, especially as the data in the paper showing that a vast majority of recorded MSNs respond to both types of cortical inputs. These data strongly suggest that both MSN types respond to both types of cortical stimulation.

Was there any attempt to explain the diversity in response by staining for D1/D2/SP/ENK to determine dMSN or iMSN identity of recorded neurons?

Another comparison would be to assess the difference between S1 and M1 responses under the same conditions. Currently, the stimulation of S1 is done indirectly by whisker deflection while M1 is activated electrically. Do the authors have comparable data showing MSN responses to electrical stimulation in S1 compared to M1? If such electrical stimuli are given with same parameters, one could conclude whether MSNs in dorsolateral striatum receive stronger or weaker inputs from these regions.

Bilateral projections: previous studies (Brown et al., Neuroscience (1996); Lei et al, J.Neuroscience (2004); Reig and Silberberg, cerebral cortex (2016)) have shown that one of the main differences between corticostriatal projections from primary sensory and motor cortices is the density of contralateral innervation. Projections to contralateral striatum from primary sensory cortex are much sparser than those from motor cortex and other more frontal regions. Do the authors have any data regarding bilateral whisker and/or motor cortex stimulation? One interesting question that could be answered using the experimental setup is the comparison between ipsilateral and contralateral electrical and sensory stimulation. The prediction, based on previous data is that such differences will be more pronounced in the sensory responses than motor stimulation.

Feedforward inhibition: the role of inhibitory connections within the striatum (mainly striatal interneurons but also MSN collaterals) was shown to be instrumental in shaping both spontaneous activity and whisker evoked responses (Reig & Silberberg, neuron 2014). Moreover, inputs to striatal interneurons (in particular FS interneurons) were shown to arise from various cortical regions, including

sensory, motor and others. This suggests that the responses described in this study (both sensory and electrical stimuli) are shaped by an inhibitory component as well. This issue should be addressed, at least in discussing the cortical inputs, and if possible also in presentation of experimental data. Such an inhibitory component could be revealed by depolarizing the MSNs recorded intracellularly.

Minor:

The authors show that 87% of recorded neurons respond to whisker stimulation. This is significantly higher than their earlier paper (2011) that showed 55% of responding neurons. What could be the reason for this difference in responsiveness? Was there any difference in the recording region or stimulus presentation?

The order of authors is different in the paper heading and in the cover pages (2nd and 3rd authors).

Results page 8: "…relatively weak Rm…". Better to use high/low Rm
